# Molecular Characterization and Functional Study of Insulin-Like Androgenic Gland Hormone Gene in the Red Swamp Crayfish, *Procambarus clarkii*

**DOI:** 10.3390/genes10090645

**Published:** 2019-08-26

**Authors:** Linlin Shi, Shuxin Han, Jiamin Fei, Long Zhang, Jonathan W Ray, Weimin Wang, Yanhe Li

**Affiliations:** 1 National Demonstration Center for Experimental Aquaculture Education, Ministry of Education, Hubei Provincial Engineering Laboratory for Pond Aquaculture, College of Fisheries, Key Lab of Freshwater Animal Breeding, Huazhong Agricultural University, Wuhan 430070, China; 2 Department of Medicine, University Hospitals Cleveland Medical Center, Case Cardiovascular Research Institute, and Harrington Heart and Vascular Institute, Case Western Reserve University, 2103 Cornell Road, Cleveland, OH 44106, USA; 3 Joan C Edwards School of Medicine, Marshall University, Huntington, WV 25701, USA; 4 Key Laboratory of Genetic Breeding and Aquaculture Biology of Freshwater Fishes, Ministry of Agriculture, Freshwater Fisheries Research Center, Chinese Academy of Fishery Sciences, Wuxi 214081, China

**Keywords:** *IAG*, sexual differentiation, RNAi, *Procambarus clarkii*, *Sxl*

## Abstract

The androgenic gland (AG) is a male-specific endocrine organ that controls the primary and secondary sexual characteristics in male crustaceans. More evidence indicates that the insulin-like androgenic gland hormone gene (*IAG*) is the key male sexual differentiation factor, particularly the application of RNA interference (RNAi) technology on *IAG*. In this study, the full-length cDNA of *IAG* (termed *PcIAG*) was isolated from the red swamp crayfish, *Procambarus* *clarkii*. Tissue distribution analysis showed that in addition to its expression in the AG of male *P. clarkii*, *PcIAG* was widely expressed in female tissues and other male tissues. The PcIAG protein was detected in the reproductive and nervous systems of adult male *P. clarkii*. Additionally, RNAi results showed that the *PcIAG* expression could be silenced efficiently, and the male sperm maturation and release possibly present a transient adverse interference at lower doses (0.1 μg/g and 1 μg/g) of PcIAG–dsRNA (*PcIAG* double-stranded RNA). Dramatically, the expression level of *PcIAG* increased sharply shortly after the injection of higher doses (5 μg/g and 10 μg/g) of PcIAG–dsRNA, which might accelerate the maturation and release of sperm. Moreover, the expression of *PcSxl* (*P. clarkii Sex-lethal*) was detected by Quantitative Real-Time PCR (qPCR) after the injection of PcIAG–dsRNA to explore whether the *PcIAG* gene regulates the *PcSxl* gene, and we found that the *PcIAG* did not directly regulate *PcSxl* in *P. clarkii*. The study could help accelerate the progress of *PcIAG* functional research and provide a useful reference for the single-sex selective breeding of *P. clarkii*.

## 1. Introduction

The decapod is the most diverse species in crustaceans, including the highly valued commercial species of the crabs, prawns, shrimps, crayfish, and lobsters [1]. Decapod aquaculture is a rapidly growing world industry that brings huge benefits for people. However, compared to vertebrates, knowledge of the sexual determination and differentiation is still poor amongst the decapod crustaceans [2]. The sexual determination mechanisms in crustaceans are diverse, and gender is plastic and affected by environmental factors [3,4]. Interestingly, in the decapods, the male sexual differentiation is more clearly defined because of the unique involvement of a male-specific endocrine gland called the androgenic gland (AG) [2]. The sexual determination effect of AG is thought to be mediated by a protein hormone named insulin-like androgenic gland hormone (IAG), which was originally known as the androgenic gland hormone (AGH), and was first isolated in isopod *Armadillidium vulgare* [5,6]. In decapods, researchers have expended much effort to purify the AG hormone without success [7]. As an alternative approach, the search for specifically *IAG*-expressed genes using a subtractive complementary DNA (cDNA) library in the decapod crayfish *Cherax quadricarinatus* has been employed [7]. Presently, more and more *IAG* cDNA sequences of decapod species have been cloned. In addition, the genome sequence of *IAG* in decapods has been isolated by genome walking based on the cDNA sequence, and their 5′-flanking regions were assayed [8]. In addition, the chemically active recombinant proteins have been synthesized based on the *IAG* cDNA sequence in crustaceans [9,10]. Generally, *IAG* is expressed exclusively in the AG of male crustaceans [11]. Nevertheless, recent studies have found that *IAG* was expressed not only in AG and other tissues of male animals, but also in female animals [12]. The mechanism of the *IAG* expression pattern is currently quite complex [13].

The red swamp crayfish, *Procambarus clarkii*, is native to south central USA and northeastern Mexico. In 1929, it was introduced into Nanjing City in China from Japan. *P. clarkii* has become an economically important freshwater species in China, and has been propagated artificially in recent years [14]. *P. clarkii* is also considered as an important crustacean model organism in research. Taketomi et al. [15] found that the AG consists of two types of cells in *P. clarkii*. The two types of cells may actually represent different secretory phases of a single type of cells. When implanted with AG or injected with the gland extracts into the early developing stage of females, the treated individuals matured with induced masculinization, which manifested primarily as the hardening of the first pair of pleopods and the inhibition of vitellogenesis [16,17]. To our knowledge, there are few documents about the *IAG* expression pattern of *P. clarkii*.

Recently, further studies by applying RNA interference (RNAi) technology have shown that the *IAG* is closely related to sexual differentiation in decapod crustaceans. In *C. quadricarinatus* intersex individuals (an active male reproductive system and male secondary sex characteristics, along with a constantly arrested ovary), *CqIAG* (*C. quadricarinatus IAG*) silencing accomplished via double-stranded RNA (dsRNA) injection induced dramatic sex-related alterations, including testicular degeneration and ovarian activation [18]. In the male *Macrobrachium rosenbergii*, *IAG*’s silence has a temporarily adverse effect on the development of secondary sexual characteristics and spermatogenesis [19]. Moreover, the fully functional sex reversal (“neofemales” shrimp) was achieved by long-term *MrIAG* silencing at PL30 [20]. These results were consistent with the effect on male secondary sexual characteristics and the sexual differentiation of *C. quadricarinatus* and *M. rosenbergii* in response to AG ablation [21,22]. Thus, the functional sex reversal could be developed via RNAi technology for the production of crustacean single-sex culture in aquaculture conditions.

Here, we obtained the cDNA sequence of the full-length cDNA of IAG isolated from the red swamp crayfish *Procambarus clarkii* (*PcIAG*) from the AG of *P. clarkii*. Regarding the encoding sequence, we analyzed the sequence structure and deduced the amino acid sequence. Then, the expression patterns of the *PcIAG* gene were analyzed in the different developmental stages and in the adult male and female *P. clarkii* at the mRNA and protein levels. In addition, we investigated the feasibility of RNA interference (RNAi) as a tool for the functional analysis of *PcIAG* and explored the change of expression level of *PcIAG* after RNAi in *P. clarkii*. Simultaneously, we also observed the expression of Sex-lethal (*PcSxl*) after *PcIAG* RNAi to determine whether *PcSxl* was regulated by *PcIAG* at a transcriptional level. These results could help accelerate the progress of *PcIAG* functional research and improve the effectiveness of RNAi in *P. clarkii*.

## 2. Materials and Methods

### 2.1. Experimental Animals and Tissue Collection

The crayfish were obtained from Luhu lake in Wuhan city, Hubei province, China. The crayfish were reared in water tanks at 20–23 °C and fed twice daily with commercial diet. The water was changed twice a week.

The fertilized eggs from gravid crayfish were cultured separately through the larval and juvenile stages at room temperature. Samples from different developmental stages within the four-month life span of *P. clarkii* were collected. The embryos were sampled in the nauplius stage (NS) and zoea stage (ZS), and the cephalothoraxes were sampled in larval stages. The gender of crayfish collected from the first to the 17th day following hatching cannot be identified, even under a dissecting microscope. The male and female crayfish 23 to 115 days after hatching were collected under anatomical lens by observing the characteristics of the first swimming foot. Tissue samples were collected from the adult male and female *P. clarkii*. Hemocytes (Hem) were collected using anticoagulant citrate dextrose solution (ACD anticoagulant) [23] and isolated by centrifugation at 800 g for 10 min at 4 °C. Tissues including the heart (Hea), hepatopancreas (Hep), stomach (St), foregut (FG), midgut (MG), hindgut (HG), antennary glands (AnGl), muscle (Mu), gill (Gi), hypodermis (Hy), eyestalk (Es), brain (Br), periesophageal nerve (PN), subesophageal ganglia (SG), thoracic ganglia (TG), ventral ganglia (VG), ovary (Ov), testis (Te), testicular ducts (TD), and androgenic gland (AG) were isolated from female and male crayfish. All the samples were immediately stored in liquid nitrogen.

This study was conducted in accordance with the ethical standards and according to the national and international guidelines and approved by the Scientific Ethic Committee of Huazhong Agricultural University (Wuhan, China) (HZAUFI-2017-012).

### 2.2. RNA Extraction and cDNA Synthesis

Total RNA was extracted from the collected tissues using TRIzol^®^ Reagent (Thermo Scientific, Waltham, MA, USA) in accordance with the manufacturer’s protocol. The isolated RNA was treated with RNase-free DNase I (Thermo Scientific, Waltham, MA, USA). Then, 1 μg of RNA was subjected to the reverse-transcription synthesis of cDNA using a ReverAid First Strand cDNA Synthesis Kit (Thermo Scientific, Waltham, MA, USA). The cDNA was stored at −20 °C for further use.

### 2.3. Amplification of the Nucleotide Sequences of PcIAG

The primers (PcIAGF: CTGCGGTAACCTGGCGGACACG; PcIAGR: GAGCAAGGCGCCGTCCTCCGG) amplifying the *PcIAG* cDNA fragment were designed based on the highly conserved nucleotides of *IAG* of the known decapod in the GenBank database. The sequences of the 5′ and 3′ ends of *PcIAG* were obtained by the rapid amplification of cDNA ends (RACE) following the SMARTer^®^ RACE 5′/3′ kit user manual (TaKaRa, Dalian, China) and a previously reported method [24]. The target fragment was ligated into the pRACE vector (used in the SMARTer^®^ RACE 5′/3′ Kit method) or pMDTM18-T vector (TaKaRa, Dalian, China) (used in the Li’ RACE method), and then transformed into *Escherichia coli* DH5α-competent cells (TaKaRa, Dalian, China). Positive clones were sequenced in the Tsingke Biological Technology Company. All the primers used in these studies are listed in Supplementary Table S1.

### 2.4. Sequence Analyses

The obtained cDNA fragment sequences were cleaned of vector and primer sequences and then assembled together, using sequence analysis software (DNASTAR Lasergene, Madison, WI, USA). The *PcIAG* cDNA full-length sequence was analyzed using the online website of the ORFfinder (https://www.ncbi.nlm.nih.gov/orffinder/) and BLAST (Basic Local Alignment Search Tool; https://blast.ncbi.nlm.nih.gov/Blast.cgi). Its deduced amino acids sequence was analyzed using the online website of ExPASy (https://web.expasy.org/compute_pi/) and DTU Bioinformatics (http://www.bioinformatics.dtu.dk/). The signal peptidase cleavage site of the signal was predicted by SignalP (http://www.cbs.dtu.dk/services/SignalP/). The putative cleavage sites were predicted by ProP 1.0 (http://www.cbs.dtu.dk/services/ProP/). In addition, the multiple sequence alignment of *PcIAG* with *IAG* peptides from 25 different crustacean species was performed by the clustalW algorithm (Supplementary Table S2), and the phylogenetic tree was constructed by the neighbor-joining method by bootstrapping 1000 replicates, using MEGA v.5.1 software.

### 2.5. Expression Pattern of PcIAG Detected by Quantitative Real-Time PCR (qPCR)

The expression pattern of *PcIAG* was determined by quantitative real-time PCR (qPCR) assays using cDNA prepared from the tissues of different developmental stages and adult male and female crayfish. The specific primers for PcIAG-qF/qR (Supplementary Table S1) were designed to generate a 170-bp fragment. qPCR was performed using a QuantStudio™ Real-Time PCR System (Thermo Scientific, Waltham, MA, USA) in a 20-μL reaction mix, containing 2 μL of cDNA template, 0.2 μL of each primer (10 μM), 7.6 μL of water and 10 μL of SYBR Premix Ex TaqTM (TaKaRa, Dalian, China). The qPCR program consisted of an initial denaturation at 94 °C for 10 min, followed by 40 cycles of 94 °C for 60 s, 64 °C for 45 s, and 72 °C for 30 s. Three replicates were set. The housekeeping genes *18S-RNA* were identical to those of *PcIAG* except that the annealing temperature was set at 59 °C. The *PcIAG* expression level was normalized to *18S-RNA* (*18S*) and calculated using the 2^−∆∆Ct^ method [25] and expressed as mean ± SD. Statistical analyses were performed using one-way ANOVA, under which differences were considered significant when *p* < 0.05 (*) or *p* < 0.01 (**).

### 2.6. Western Blotting

Tissue samples were lysed in RIPA (Radio Immunoprecipitation Assay) Lysis Buffer (YEASEN, Shanghai, China) and centrifuged at 12,000× *g* at 4 °C. The protein concentration was determined using the BCA Protein Quantification Kit (YEASEN, Shanghai, China). Protein samples were separated in tris-glycine SDS-PAGE (12%) gel and transferred onto PVDF (polyvinylidene fluoride) membranes (300 mA for 20 min). The membrane was blocked overnight at 4 °C with 5% nonfat milk in TBST (Tris-Buffered Saline Tween-20), and then incubated for 2 h at room temperature with anti-PcIAG polyclonal antibody (prepared by Dia-An Biotech, Inc., Wuhan, China) (1:1000 dilution) targeting the full-length *PcIAG* ORF (Open Reading Frame) sequence, and anti-β-tubulin rabbit polyclonal antibody (Abcam, London, UK) (1:5000 dilution). After washing three times in PBST (phosphate buffered solution containing Tween-20), the membrane was incubated with HRP-conjugated goat anti-rabbit secondary antibodies (LI-COR, Lincoln, NE, USA) (1:10000 dilution) at room temperature for 2 h in the dark. After washing, the membrane was analyzed using an Odyssey Infrared Imaging System (LI-COR, Lincoln, NE, USA).

### 2.7. Histology of the AG in P. clarkii

Tissue samples were fixed in 4% paraformaldehyde and transported to Wuhan Servicebio Technology Co., LTD for further processing according to conventional procedures. The 5-μm sections were placed on glass slides and stained with hematoxylin and eosin. Sections (5-μm thick) of each sample were sliced from each block, mounted on glass slides, deparaffinized in xylene, rehydrated through a series of decreasing alcohol concentrations, and stained with hematoxylin and eosin (H&E). The slices were examined and photographed under a Nikon Eclipse E100 (Nikon, Tokyo, Japan) light microscope with a video camera.

### 2.8. RNAi

DNA fragments of the *PcIAG* and enhanced green fluorescent protein gene (*EGFP*) were amplified by specific primers, which had a T7 promoter at their 5′ ends (as listed in Supplementary Table S1), and used as templates for dsRNA (double-stranded RNA) synthesis. PcIAG–dsRNA and EGFP–dsRNA (used as control) were synthesized using a TranscriptAid T7 High Yield Transcription kit (Thermo Fisher Scientific, Waltham, MA, USA) according to the manufacturer’s instructions and purified by phenol (pH 4.7): chloroform extraction and ethanol precipitation, and then dissolved in 20 μL of diethypyrocarbonate (DEPC)-treated water. The quality and quantity of dsRNAs were measured using 1% gel electrophoresis and a Nanodrop 2000 (Thermo Fisher Scientific, Waltham, MA, USA). The dsRNA was maintained at −20 °C until used.

For the *PcIAG* silencing experiments, the adult male crayfish were classified into eight groups (*n* = 25 each) as follows: four groups were injected with PcIAG–dsRNA at different doses 0.1 μg, 1 μg, 5 μg, and 10 μg dsRNA/g body weight from the crayfish’s tail abdomen muscle, the other four groups were injected with GFP–dsRNA according to PcIAG–dsRNA doses.

Tissues were surgically obtained at five time points 1 day, 3 days, 5 days, 7 days, 10 days, and 14 days post dsRNA injection (dpi), and immediately preserved in liquid nitrogen. Nine tissues including Te, TD, AG, Es, Br, PN, SG, TG, and VG (Supplementary Figure S1) were collected from three individuals of each group at the same time point. Total RNA was extracted from the collected various tissues, and the reverse-transcribed cDNA was kept at −20 °C until use.

For checking the RNAi effect, the expression level of *PcIAG* was determined by qPCR. The qPCR conditions of *PcSxl* and the housekeeping gene *18S-RNA* were identical to those of *PcIAG* except that the annealing temperature was set at 59 °C. The *PcIAG* and *PcSxl* expression levels were normalized by *18S-RNA* and calculated using the 2^-∆∆Ct^ method. All the data were given in terms of relative mRNA expressed as mean ± SD. Statistical analyses were performed using one-way ANOVA by the least significant difference (LSD) multiple-range test [26], under which differences were considered significant when *p* < 0.05. Figures were created using GraphPad Prism 7.0 (GraphPad Software, San Diego, CA, USA). In addition, the structures of testis and testicular ducts at the 14th day after dsRNA injection were observed by histological examination.

## 3. Results

### 3.1. Characterization of the AG in P. clarkii and Sequence Analysis of PcIAG cDNA

**Figure 1:**
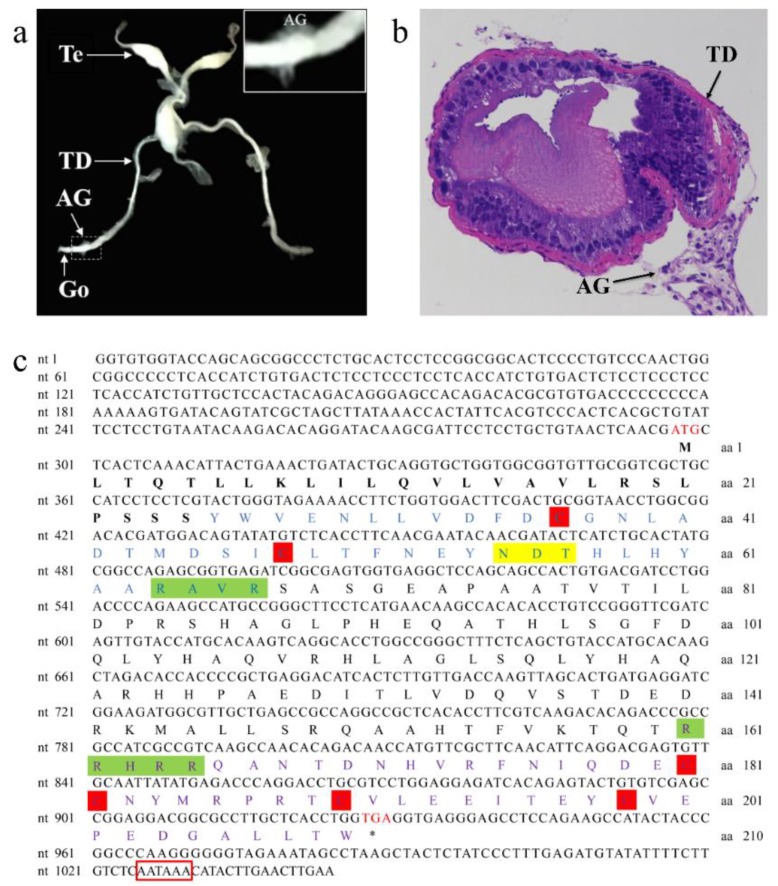
Identification of the androgenic gland and prediction of the full-length cDNA of *IAG* isolated from the red swamp crayfish *Procambarus clarkii* (*PcIAG*) structure in male *P. clarkii*. (**a**) Anatomical map of the mature male reproductive system of *P. clarkii*. The reproductive system includes testis (Te), testicular ducts (TD), and gonopores (Go). The androgenic gland (AG) is attached to the subterminal region of the testicular ducts. The photo in the upper right corner is magnification of the dashed frame. (**b**) Histological section of the TD and AG of a mature *P. clarkii* male (×200). A 5-μm thick cross-section of the AGs and TD tissues stained with hematoxylin and eosin. (**c**) Nucleotide sequence and deduced amino acid sequence of *PcIAG* cDNA. The nucleotide sequence number is on the right, and the deduced amino acid sequence number is on the left side of the figure. The start codon (ATG) is marked with red, and the stop codon (TGA) is marked with red and indicated with “*”. A polyadenylation signal (AATAAA) is marked with a red box in the 3ʹ untranslated region (UTR). The putative signal peptide sequence is shown in bold. The putative B, C, and A chains are marked with blue, black, and purple, respectively. The six conserved cysteine residues are marked with red shading. Two predicted arginine C proteinase cleavage sites (R_67_/S_68_ and R_165_/Q_166_) are highlighted with green shadings, while a predicted *N*-glycosylated site is marked with yellow shading.

The AG of *P. clarkii* is situated in the base of the last pair of walking legs, similar to other crayfish species such as *P. fallax* and *C. quadricarinatus*. The male reproductive system of *P. clarkii* is composed of the testis (Te), testicular duct (TD), and gonopores (Go). The gland is located upstream of the Go, and is loosely and unevenly attached around the outer wall of the subterminal TD (Figure 1a,b).

The nucleotide and deduced amino acid sequences of *PcIAG* cDNA are shown in Figure 1c. The full-length *PcIAG* cDNA is 1047 bp, with a 633-bp open reading frame (ORF), a 5′ untranslated region (296 bp), and a 3′ untranslated region (118 bp) tailed with the putative polyadenylation site AATAAA. The deduced protein of *PcIAG* cDNA is 210 aa, with a predicted mass of 23.82 KDa and theoretical isoelectric point (pI) of 5.86. A signal peptidase cleavage site is between the S25 and Y26, as predicted by SignalP software. Two putative cleavage sites between R67 and S68, and R165 and Q166 were predicted by software ProP 1.0. Therefore, the truncated PcIAG proteins contain: a signal peptide (M1-S25, 25 aa), B chain (Y26-R67, 42 aa), C chain (S68-R165, 98 aa), and A chain (Q166-W210, 45 aa). A potential N-linked glycosylation motif (N55-D56-T57) was observed in the B chain, and the T55 residue was likely glycosylated, and six conserved Cys residues were observed in the B chain and A chain.

### 3.2. Multiple Alignment and Phylogenetic Tree Analysis of P. clarkii

**Figure 2:**
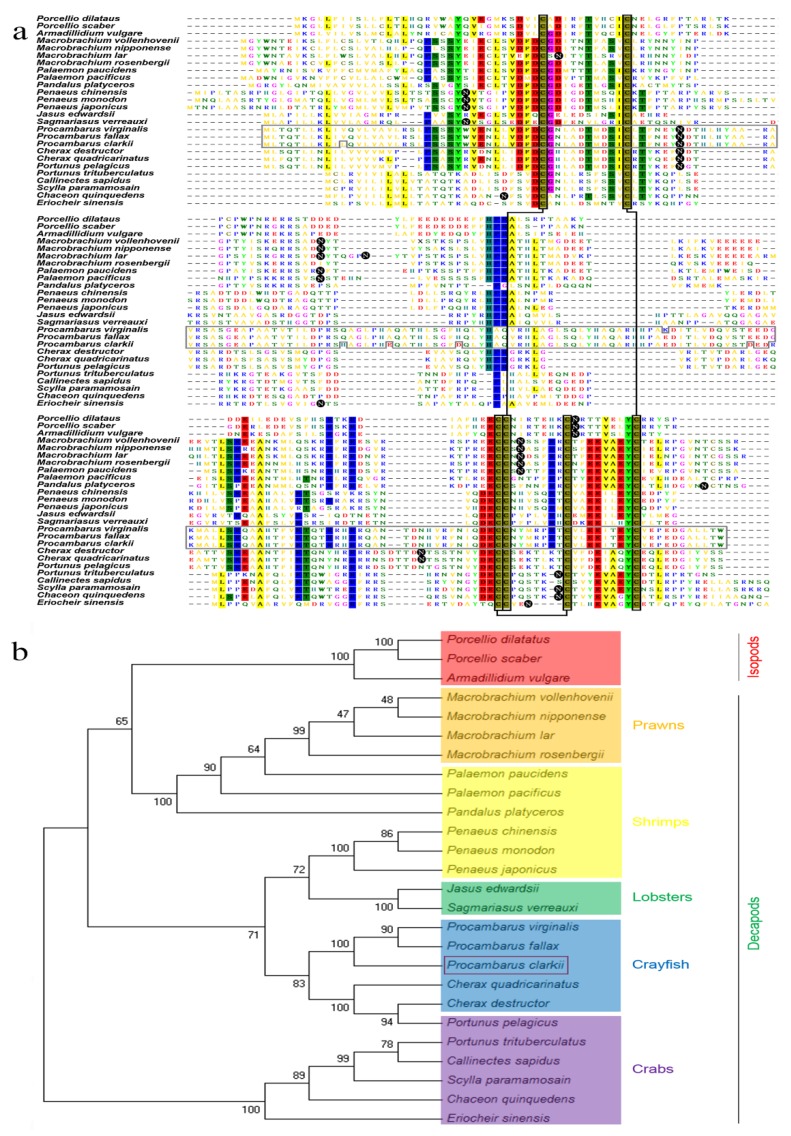
Multiple sequence alignment and phylogenetic tree analysis of deduced insulin-like androgenic gland hormone gene (*IAG*) amino acid sequences in 26 crustacean species. The sequences of 23 decapod species and three isopod species are listed in Table S2. (**a**) Multiple sequence alignment. The alignment was performed by the clustalW algorithm. The identical residue sites in regions with more than a 50% conserved level are highlighted with a colored square background, while the predicted N-glycosylation sites are highlighted with a black circular background. The backbones of the six conserved cysteine residues are marked with black boxes, which give rise to three disulfide bridges (lines connecting the black boxes). The homologous region of the three species of the genus *Procambarus* is marked with a gray box. (**b**) Phylogenetic tree. Based on the clustalW algorithm alignment of 26 crustacean species, the phylogenetic tree was constructed by the neighbor-joining method with bootstrapping 1000 replicates, using MEGA 5.1 software. The isopod species are highlighted with red background. Prawns, shrimps, lobsters, crayfish, and crabs of the decapods are highlighted with the orange, yellow, green, blue, and purple backgrounds, respectively. *P. clarkii* is marked with a box.

A multiple sequence alignment of AG-specific insulin-like peptides for three isopods and 23 decapods are shown in Figure 2a, revealing the poor homology of this family. The predicted positions of only seven amino acids, including six cysteine residues, were conserved in the 26 species. However, the predicted three disulfide bridges (two inter-chain bonds and one intra-chain bond) produced by the six conserved cysteine residues are pivotal for the functional structure of the mature dimeric peptide in the insulin family of hormones. The phylogenetic tree shows that the IAG protein is highly conserved among crab species, including *Portunus trituberculatus*, *C. quinquedens*, *Eriocheir sinensis*, *Scylla paramamosain*, and *Callinectes sapidus*. Curiously, the *P. pelagicus* sequence demonstrates more similarity to *C. destructor* than *P. trituberculatus*. The shrimps are divided into two groups: one group (*Palaemon* and *Pandalus* genus) gathers with the prawns, and the other group (*Penaeus* genus) gathers with the lobsters. The three isopods and the prawns and some shrimps of decapod crustaceans were grouped together, while the crayfish and lobsters come out in the same vein. Comparative analysis of the *IAG* coding region among the three *Procambarus* species shows that the amino acid sequence of the *P. clarkii* is highly similar to the corresponding sequence in the two species *P. virginalis* and *P. fallax* (Figure 2).

### 3.3. Expression Profile of PcIAG Gene in Different Developmental Stages of P. clarkii and Adult Crayfish

**Figure 3:**
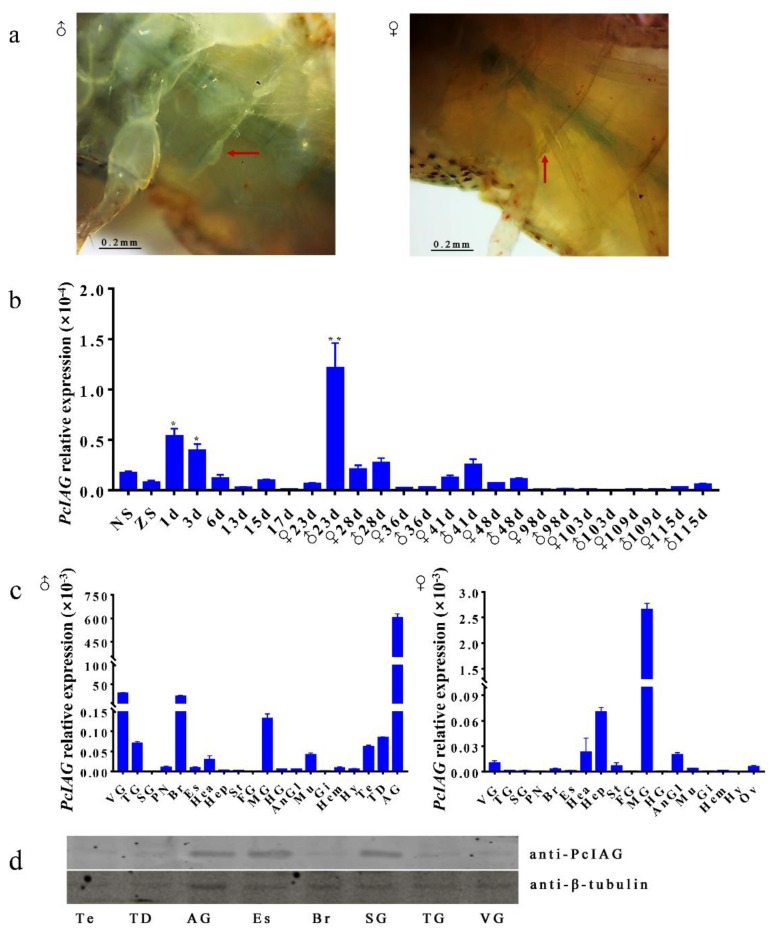
Expression pattern of *PcIAG* gene and Western blot analysis of the PcIAG protein. M, DNA marker; NC, negative control; VG, ventral ganglia; TG, thoracic ganglia; SG, subesophageal ganglia; PN, periesophageal nerve; Br, brain; Es, eyestalk; Hea, heart; Hep, hepatopancreas; St, stomach; FG, foregut; MG, midgut; HG, hindgut; AnGl, antennary glands; Mu, muscle; Gi, gill; Hem, Hemocytes; Hy, hypodermis; Ov, ovary; Te, testis; TD, testicular ducts; AG, androgenic gland. (**a**) The first swimming foot of the *P. clarkii* at the 23rd day after hatching. The first swimming foot of the young male and female *P. clarkii* is indicated by the red arrow. (**b**) Expression pattern of *PcIAG* at different developmental stages within four months of *P. clarkii*, *p* < 0.05 (*), *p* < 0.01 (**). (**c**) Expression pattern of *PcIAG* gene in adult crayfish. (**d**) Western blot analysis of the PcIAG protein in adult male *P. clarkii*. SG, subesophageal nerve (with periesophageal nerve); other abbreviations represent the same tissue name as above.

The first and second molting of the larval crayfish were at the third and sixth day after hatching. Using the dissecting microscope, we found that the male external characteristics (the base bud of the first swimming foot) of the *P. clarkii* appeared on the 23rd day following hatching (Figure 3a).

The expression pattern of *PcIAG* at different developmental stages was analyzed by qPCR (Figure 3b). The results indicated that the *PcIAG* expression level has two peaks: one was from the first to third day after hatching, and the other was at the 23rd day after hatching. In addition, the expression levels of *PcIAG* remained dramatically low from the 98th to the 109th day after hatching, regardless of gender. The reduction in expression is probably due to the gradually concentrated expression of *PcIAG* in AG tissues and the extremely low *IAG* mRNA in cephalothorax during the crayfish development.

The cDNA samples obtained from three adult pairs of male and female crayfish tissues were used for qPCR amplification to examine the tissue expression of the *PcIAG* transcript. The results of fluorescence qPCR detected *PcIAG* mRNA expression in 20 male tissues and 18 female tissues. The expression level of *PcIAG* was the highest in AG followed by VG and Br in males (Figure 3c).

The expression of the PcIAG protein was selectively determined in the tissues involved in the reproductive and nervous systems of adult male *P. clarkii*, including Te, TD, AG, Es, Br, SG (with PN), TG, and VG. The results showed the expression between mRNA and protein in the detected tissues involved in the nervous system are not consistent, while the *IAG* mRNA and protein expressed in the male reproductive system including Te, TD, AG tissue present similar profiles (Figure 3d).

### 3.4. The PcIAG Gene Expression Profile in AG after RNAi

DNA fragments of the *PcIAG* (418 bp) and *EGFP* (567 bp) were amplified by specific primers with the T7 promoter sequence (Supplementary Figure S2). PcIAG–dsRNA and EGFP–dsRNA were synthesized using the specific DNA fragment by in vitro transcription (Supplementary Figure S2a).

**Figure 4:**
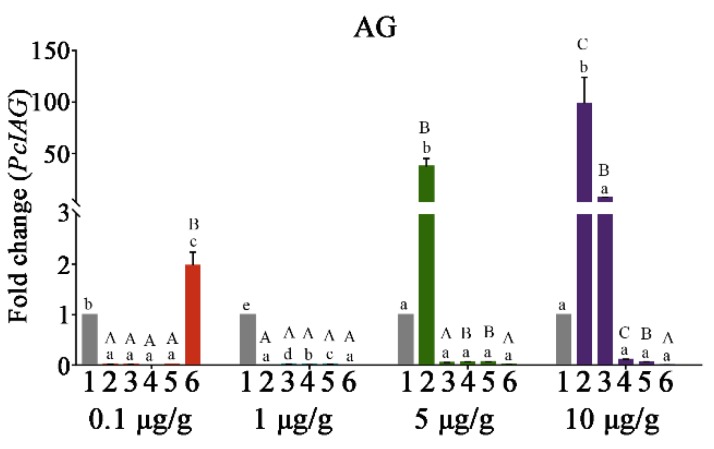
The change of *PcIAG* gene expression level in AG tissue at four doses of PcIAG–dsRNA (*PcIAG* double-stranded RNA). Column “1” in the figure represents the *PcIAG* expression after the injection of enhanced green fluorescent protein gene (EGFP)–dsRNA. Columns “2”, “3”, “4”, “5”, and “6” in the figure represent the *PcIAG* expression at different time points 1, 3, 7, 10, and 14 days post dsRNA injection (dpi) after the injection of PcIAG–dsRNA, respectively. Different lowercase letters indicated significant differences among 1, 3, 7, 10, and 14 dpi for the same PcIAG–dsRNA concentration. Different capital letters indicated significant differences among PcIAG–dsRNA dose groups at the same time point (*p* < 0.05).

**Figure 5:**
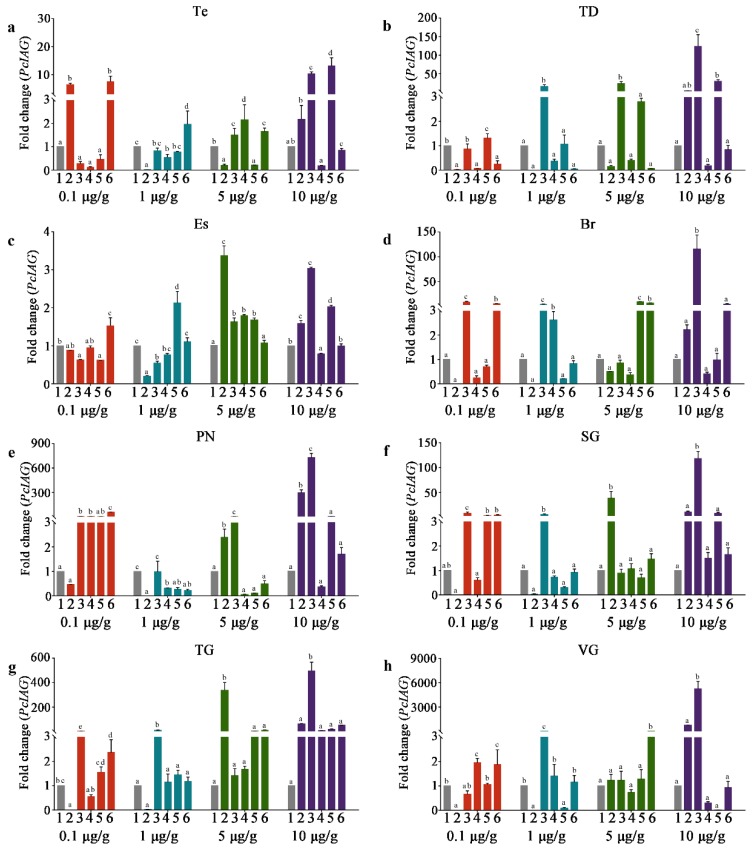
The change of *PcIAG* gene expression level in tissues at four doses of PcIAG–dsRNA. Column “1” in the figure represents the *PcIAG* expression after the injection of EGFP–dsRNA. Columns “2”, “3”, “4”, “5”, and “6” in the figure represent the *PcIAG* expression at different time points: 1, 3, 7, 10 and 14 dpi after the injection of PcIAG–dsRNA, respectively. (**a**) Testis (Te); (**b**) Testicular ducts (TD); (**c**) Eyestalk (Es); (**d**) Brain (Br), (**e**) Periosophageal nerve (PN); (**f**) Subesophageal ganglia (SG); (**g**) Thoracic ganglia (TG); (**h**) Ventral ganglia (VG). Different lowercase letters indicated significant differences among 1, 3, 7, 10, and 14 dpi for the same dsRNA concentration (*p* < 0.05).

Based on the simplified analyses method explored for evaluating the effect of RNAi shown in Supplementary Figure S2d,e, it was feasible and simplified to evaluate the effect of RNAi by calculating the trend of the *PcIAG* expression fold change relationship between the same concentration of the PcIAG–dsRNA group and the EGFP–dsRNA group at the same time point, and the results shown in Figure 4; Figure 5.

In AG, the expression of *PcIAG* was shown in Figure 4. In the 0.1 μg/g PcIAG–dsRNA dose group, the expression level of *PcIAG* was significantly down-regulated at 1 dpi in contrast with the control group, and this expression level continued until 10 dpi, while the expression level was rapidly up-regulated at 14 dpi and significantly higher than the control group. In the 1 μg/g PcIAG–dsRNA dose group, the expression level of *PcIAG* was significantly down-regulated within 1 dpi to 14 dpi in contrast with the control group, and presented the lowest level of *PcIAG* expression at 1 dpi and 14 dpi. The interference efficiency was not less than 99% in the 0.1 μg/g dose group (during the period 1 to 10 dpi) and 1 μg/g dose group (during the period 1 to 14 dpi).

In the 5 μg/g PcIAG–dsRNA dose group, the expression level of *PcIAG* was significantly up-regulated at 1 dpi in contrast with the control group, and went down after that, but not significantly compared with the expression level of the control group. In the 10 μg/g PcIAG–dsRNA dose group, the change in the expression of *PcIAG* was similar to that of the 5 μg/g dose group. The expression level of *PcIAG* still maintains signs of up-regulation at 3 dpi and then went down after that, although the expression level of *PcIAG* from 3 dpi to 14 dpi was not significant compared to the control group.

### 3.5. The PcIAG Gene Expression Profile in Other Tissues after RNAi

In other tissues, the expression of *PcIAG* at different time points after the injection of four doses of PcIAG–dsRNA was shown in Figure 5. In the Es and PN, the expression level of *PcIAG* was significantly down-regulated for a period of time after the injection of 1 μg/g of PcIAG–dsRNA, although the interference efficiency was weaker than that of AG. However, in PN (at the 0.1 μg/g, 5 μg/g, and 10 μg/g doses), Es (at the 5 μg/g and 10 μg/g doses), SG (at the 10 μg/g dose), and TG (at the 5 μg/g and 10 μg/g doses), the expression of *PcIAG* showed an up-regulated change after the injection of the PcIAG–dsRNA, indicating that nerve tissues also have a stress response to PcIAG–dsRNA, and the sensitivity may be higher and the effect time longer compared with AG.

### 3.6. The Expression Profile of PcSxl after RNAi

**Figure 6:**
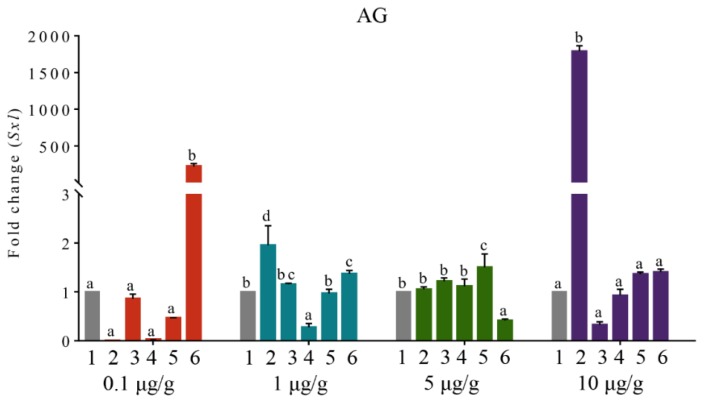
The change of *PcSxl* gene expression level in AG tissue at four injection doses of PcIAG–dsRNA. Column “1” in the figure represent *PcSxl* expression after the injection of EGFP–dsRNA. Columns “2”, “3”, “4”, “5”, and “6” in the figure represent the *PcSxl* expression at different time points: 1, 3, 7, 10, and 14 dpi after injection of PcIAG–dsRNA, respectively. Different letters indicated significant differences among 1, 3, 7, 10, and 14 dpi for the same dsRNA concentration (*p* < 0.05).

In AG, the expression of *PcSxl* at different time points after the injection of four doses of PcIAG–dsRNA was shown in Figure 6. In the 0.1 μg/g PcIAG–dsRNA dose group, the expression level of *PcSxl* was not significantly different within 1–10 dpi in contrast with the control group, while the expression level was rapidly up-regulated at 14 dpi and significantly higher than the control group. In the 1 μg/g PcIAG–dsRNA dose group, the expression level of *PcSxl* was significantly up-regulated at 1 dpi and 14 dpi in contrast with the control group, and the expression level was significantly down-regulated at 7 dpi, while the expression level was not significantly different from the control group at 3 dpi and 10 dpi. In the 5 μg/g PcIAG–dsRNA dose group, the expression level of *PcSxl* was significantly up-regulated at 10 dpi in contrast with the control group, while the expression level was significantly down-regulated at 14 dpi. At the 10 μg/g PcIAG–dsRNA dose group, the expression level of *PcSxl* was similar to that of *PcIAG*, which was significantly up-regulated only at 1 dpi. However, as the injection time prolonged, the expression trend of *PcSxl* was opposite to that of *PcIAG*, although the expression levels of *PcSxl* and *PcIAG* at each time point was not significant compared with the control group. In brief, no obvious regularity was found regarding the change in the expression profile of *PcSxl*. In addition, the changes of *PcSxl* expression in other tissues were also irregularly observed (Figure S3). Thus, it might be determined that *PcIAG* did not directly regulate *PcSxl* in *P. clarkii*.

### 3.7. The Gonadal Development after Injection of dsRNA

**Figure 7:**
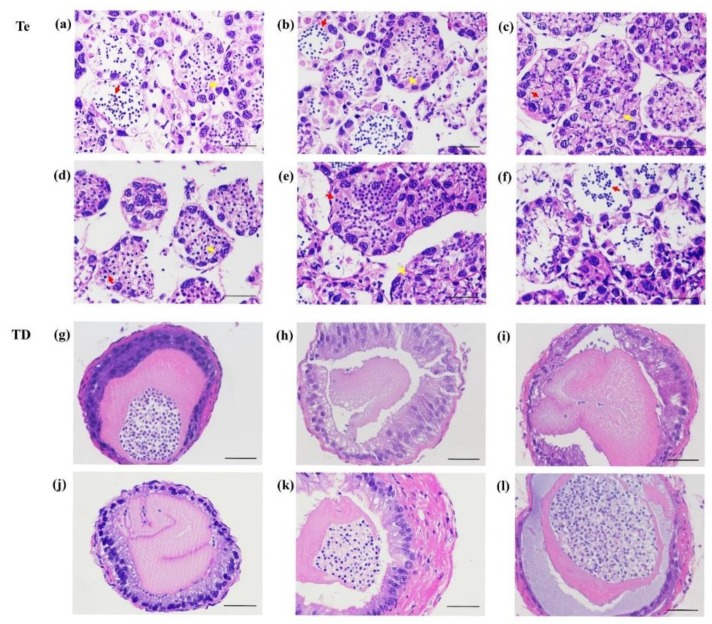
Effect of PcIAG–dsRNA on the testis development of *P. clarkii*. Hematoxylin and eosin (H&E)-stained cross-sections used for structure description. Te, Testis (**a**–**f**); TD, Testicular ducts (**g**–**l**); (**a&g)**, 1 μg/g of EGFP–dsRNA (low-dose control group); (**b**,**h**) 1 μg/g of PcIAG–dsRNA; (**c**,**i**) 0.1 μg/g of PcIAG–dsRNA; (**d**,**j**) 5 μg/g of EGFP–dsRNA (high-dose control group); (**e**,**k**) 5 μg/g of PcIAG–dsRNA; (**f**,**l**) 10 μg/g of PcIAG–dsRNA. Mature sperm cells were indicated by red arrows. Immature sperm cells were indicated by yellow arrows.

Through histological observation of the testis and testicular ducts of *P. clarkii* on the 14th day after dsRNA injection, we found that most sperm cells in the testis from the 0.1 μg/g PcIAG–dsRNA injection group were still immature (Figure 7c); simultaneously, the mature sperm in the spermatophores was poor compared with the control group (Figure 7i). In the 1 μg/g PcIAG–dsRNA injection group, the testicular development was similar to that of the control group, with more mature sperm cells (Figure 7b); however, it is hard to find mature sperm in spermatophores (Figure 7h). In the 5 μg/g PcIAG–dsRNA injection group, the testis was filled with plump sperm cells (Figure 7e), and the spermatophore was stuffed with a sperm cluster (Figure 7k). In the 10 μg/g PcIAG–dsRNA injection group, the mature sperm was released from the testis, causing greater lacunae in the spermatogenic acini, and a bigger sperm cluster in the spermatophore, compared to the 5 μg/g PcIAG–dsRNA injection group (Figure 7f,l). The results indicated that PcIAG–dsRNA at low doses (0.1 and 1 μg/g) may impede the maturation of sperm cells and inhibit the release of mature sperm, while PcIAG–dsRNA at high doses (5 μg/g and 10 μg/g) may accelerate maturation and the release of sperm.

## 4. Discussion

AG is a male-specific endocrine organ that controls male primary and secondary sexual characteristics. In the past decades, the gland function has been investigated in a number of crustacean species [27]. Recently, more evidence indicates that its secreted insulin-like androgenic gland hormone gene (*IAG*) is a key mechanism of male sexual determination. *P. clarkii* has become one of the most economically important aquaculture species in China. Also, the creature has been broadly used as a studying model in many research fields including animal behavior, evolution, viral infection, environmental stress, and toxicity. As sequencing technology develops, the transcriptome and genome data of *P. clarkii* are also being explored by researchers. However, understanding of the molecular mechanisms involved in the sexual regulation of this species is limited. Here, we describe *IAG* transcripts in *P. clarkii* AG, and are the first to report the expression of *IAG* in various tissues of *P. clarkii*.

A 1047-bp *PcIAG* cDNA sequence was obtained. Compared with the *P. clarkii IAG* cDNA sequence (854 bp, KT343750.1) deposited in GenBank in 2015 by Savaya-Alkalay et al., we further determined the upstream 5ʹ UTR sequence, which is 213 bp. The predicted peptide of *PcIAG* was cleaved into the B chain, C chain, and A chain (the order is similar to the insulin family peptides) by two putative cleavage sites. The proteolysis of larger inactive precursors at sites labeled by the consensus sequence (R-X-K/R-R) is a common step to produce active peptide hormones [28]. Thus, it is difficult to successfully purify AG hormones in decapods. In addition, a potential N-linked glycosylation motif was observed in the B chain, which is a common post-translational marker that regulates protein function and activity as well as biodiversity [29]. The multiple sequence alignment and phylogenetic tree analysis of deduced *IAG* amino acid sequences in 26 crustacean species showed that the IAG is highly conserved among crayfish. Especially, the deduced amino acid sequence of *P. clarkii* is different from the corresponding sequences in two species of *P. fallax* and *P. virginalis* with only a few amino acid residues. The possible explanation is that the ancestors have the same origin [12], and the cumulative evolutionary pressure is insufficient to make a significant change in the gene. *P. virginalis* is a relatively new species that probably recently diverged from *P. fallax* [30]. Another possible explanation for *P. virginalis* is that the reproductive strategy of an all-female clone [31] disregards the *IAG*. Interestingly, the prawns and some shrimps of decapods are closer to the ones of isopods than to the ones of other decapods, indicating that the evolutionary process of the decapods and isopods was very complicated.

The *IAG* from AG is known to regulate male sexual differentiation in decapod crustaceans [18,27]. Early studies reported that *IAG* was only expressed in male AG [7]. However, the expression of *IAG* is detectable in many other tissues in addition to AG. It has been reported that the expression is detected in other tissues, such as the ovary, testis muscle, and hepatopancreas [12,32,33]. The insulin family signal pathway is essential for normal growth and development [34]; the expression of *PcIAG* in the muscle and digestive system of the adult male and female *P. clarkii* may play a regulatory role in the growth and development of the organisms. More recent studies show that *IAG* is also expressed in the nervous system [17]. However, the transcriptional level of *MnIAGBP* was unaffected by *MnIAG* dsRNA injection in the tissues, including the eyestalk, brain, and nerve cord [35]. The role of *IAG* in the nervous system needs to be further studied. Although widely expressed in many tissues, the *PcIAG* expression in AG was much higher than in other tissues, suggesting the dominant role of *IAG* in sexual determination.

The expression of *IAG* in early developmental stages may be marked as the initiating point of sexual differentiation. In *M. rosenbergii*, *IAG* was expressed from as early as 20 days after metamorphosis, prior to the appearance of external male sexual characteristics (in the time range of PL20 to PL120) [27], and full functional sex reversal is achieved by silencing the *IAG* at PL30 [20]. In *Litopenaeus vannamei*, the genital organ was fully recognized at PL16, and the external sexual differentiation of female and male was recognized at PL50 [36]. In this article, the expression level of *PcIAG* reaches a peak from the first to third days after hatching, and another higher expression peak appeared in the larva with male external characters at the 23rd day after hatching. The sexual differentiation of *P. clarkii* may begin from the first to third day after hatching, which is earlier than the complete metamorphosis prawns or shrimps. Further studies are required to prove the prediction.

RNA interference (RNAi) is an ancient, ubiquitous, and evolutionarily conserved mechanism for sequence-specific post-transcriptional gene silencing (PTGS) in many eukaryotes [37]. The discovery of the RNAi phenomenon has revolutionized functional genomics research in many fields [19,38,39,40]. In aquaculture, RNA interference is the most recent tool against viral diseases in shrimp, and it is deemed as a promising biotechnology to boost shrimp production [41]. In addition, the application of RNAi technology in the *IAG* has made a breakthrough in the sexual determination and sexual differentiation research of some economic crustaceans. In this study, RNAi was first used as a tool to investigate the function of *PcIAG* in *P. clarkii*.

In Lepidoptera, a “standard” range of amounts of dsRNA is injected, which varies between 1–100 mg. Since the same range is routinely injected in small and large species, the calculated sensitivity to RNAi appears lower in small species [42]. Similar phenomena exist in crustaceans [25,43,44,45]. Therefore, establishing a clear dose-to-interference response and determining the optimal interference dose and timing are critical to the success of RNAi technology. In *M. rosenbergii*, the *MrIAG* interference-dependent dose was found to be more effective in the 1 μg/g and 5 μg/g dose groups on day 7 after dsRNA injection, while the interference effect was not obvious in the 0.1 μg/g dose group. The time dependence of interference in the 5 μg/g dose group was explored, and the interference was found to be optimal on days 3 and 7 after dsRNA injection [20]. In *P. clarkii*, the low doses (0.1 μg/g and 1 μg/g) of the PcIAG–dsRNA successfully interfered with the *PcIAG* expression of AG, and the interference effect of the 1 μg/g dose was more durable than that of the 0.1 μg/g dose (0.1 μg/g dose at 1–10 dpi, 1 μg/g dose at 1–14 dpi). However, the high doses (5 μg/g and 10 μg/g) of the PcIAG–dsRNA had a counterproductive effect on interfering with *PcIAG* expression. Furthermore, the effect of different doses of PcIAG–dsRNA within 14 days after injection was further confirmed by histology.

Choosing the best functional tissue selection is an essential factor for the success of RNAi. In our laboratory, it was found that the *PcIAG* of *P. clarkii* was mainly expressed in AG and was also widely expressed in other tissues. Therefore, this study also analyzed the change of the *PcIAG* gene expression level in tissues of the reproductive and nervous systems at four doses of PcIAG–dsRNA. Although the expression levels of *PcIAG* in Es and PN were significantly down-regulated for a period of time after the injection of 1 μg/g of PcIAG–dsRNA, the interference effect was much lower than that of AG, regardless of efficiency or duration. The result indicated that AG is the best choice for the functional tissue of *PcIAG* RNAi. Functional studies of *PcIAG* genes have shown their roles in testicular development and spermatogenesis. In *M. rosenbergii*, sperm was absent in either the testis or sperm duct after the silencing of *PcIAG* by dsRNA injection [19]. In *C. quadricarinatus*, the silencing of *PcIAG* led to a reduction in sperm production [18]. In this study, the results suggest that the *IAG* of *P. clarkii* may play an important role in sperm maturation and release. When the *PcIAG* expression of *P. clarkii* was silenced, the sperm maturation and release were inhibited in the testis, and the sperm cluster was hard to locate in the spermatophore. Conversely, when *PcIAG* expression was up-regulated due to the injection of high doses of PcIAG–dsRNA, sperm maturation and release were accelerated in the testis, and enriched in the spermatophore.

Furthermore, an interesting phenomenon was discovered in this study. In AG, the expression of *PcIAG* from 3 dpi to 10 dpi was significantly up-regulated compared to 1 dpi and 14 dpi at the 1 μg/g dose of PcIAG–dsRNA, while the interference efficiency at these points was not less than 99%. In the high doses of PcIAG–dsRNA, the expression of *PcIAG* was significantly up-regulated, and as the dose increased, the larger the fold of up-regulated at 1–10 dpi. Similar phenomena have been found in other tissue such as PN, Es, SN, and TN. The authors speculate that as the dose of PcIAG–dsRNA increases, more and more intense stress reactions may be produced in the crayfish body. In the study of zebrafish gene function, as the dsRNA dose increased, the efficiency of each specific phenotype was increased, while the efficiency of inducing gene-specific defects of the fertilized egg was also increased [46]. From this point of view, the increase in the dose of dsRNA was not an absolute advantage for the success of RNAi. Nevertheless, the range of dsRNA fragments covered the fragment of qRT-PCR (Table S1). It is possible that the high level of *IAG* mRNA detected at 1 dpi (5 µg/g) and at 1 and 3 dpi (10 µg/g) comes from the remaining intact injected dsRNA, which is reverse transcribed (high doses), whereas the endogenous *IAG* mRNA is degraded. Again, the high level observed at 14 dpi (0.1 µg/g) is likely due to a decrease of the dsRNA effect (low doses). That means that it is possible that the intact injected PcIAG–dsRNA remains in the body such that the level of *PcIAG* expression detected is higher than the actual level. Further studies are needed to be clarify the inference veracity.

The regulation of sexual determination signaling in insect somatic cells is constituted by a cascade involving several genes. In Drosophila melanogaster, the cascade contains four main genes beginning with *Sxl* on top, Transformer (*Tra*), and Transformer-2 (*Tra-2*) in the middle, and Doublesex (*Dsx*) on the bottom [47]. Crustaceans are believed to be the ancestors of insects [48]. Currently, these genes—which are homologous to the sexual determination—have been found in many crustaceans [49,50,51,52]. Studies have shown that *Dsx* might be the upstream regulator of *IAG*. *MrDmrt11E* RNAi induced a significant decrease of the transcript of *MrIAG* in *M. rosenbergii* [53]. In *Fenneropenaeus chinensis,* the knockdown of *FcDsx* could reduce the expression of *FcIAG*. Simultaneously, a putative *FcDsx* binding site was identified on the promoter region of *FcIAG* [51]. Recent studies showed that the *Sxl* gene might play an important role in the embryonic development and gametogenesis of prawn or shrimp, even in sex differentiation, but not in sex determination [49,54]. Curiously, whether *Sxl* was regulated by *IAG* at a transcriptional level, or not has yet to be determined. In the present study, there was no direct regulation relationship found between the *PcIAG* and *PcSxl* of *P. clarkii*, while the expression of *PcSxl* was not affected regularly by the injected PcIAG–dsRNA. However, whether the gene *PcSxl* regulates *PcIAG* in *P. clarkii* needs further study in the future.

## 5. Conclusions

In summary, we confirmed that the AG of *P. clarkii* is in the typical region (the base of the male last pair of walking legs attaching the outer wall of the subterminal TD) and obtained the *PcIAG* cDNA sequence from the AG of the male *P. clarkii*. We found that the tissue distribution of *PcIAG* in adult male and female tissues was complex. While highest in AG, the expression of *PcIAG* was widely detected in multiple tissues. The male external characteristics of *P. clarkii* were found on the 23rd day after hatching, and the *PcIAG* expression level was robustly elevated at this time point. Additionally, we successfully established a specific long double-stranded RNA (PcIAG-dsRNA) that was capable of effectively interfering with the *PcIAG* gene of *P. clarkii*, with which the function of *PcIAG* was initially explored.

## Supplementary Materials

The following are available online at https://www.mdpi.com/2073-4425/10/9/645/s1, Table S1. Primers used in this experiment. Table S2. Annotation of the IAG used in the present study. Figure S1. Anatomical map of the nervous system of *P. clarkii*. Figure S2. Detection of the dsRNA quality, DNA fragments and simplified analyses method explored for evaluating the effect of RNAi. Figure S3. The change of *PcSxl* gene expression level in tissues at four doses of PcIAG-dsRNA.
