# Supplementary material for: Molecular Characterization and Functional Study of Insulin-Like Androgenic Gland Hormone Gene in the Red Swamp Crayfish, Procambarus clarkii
Source: Genes (Basel). 2019 Aug 26;10(9):645. doi: 10.3390/genes10090645 (PMC6770367; doi:10.3390/genes10090645)
Supplement: Supplementary file 1 [file genes-10-00645-s001.zip › Supplementary Materials/Figure S1.docx]

**
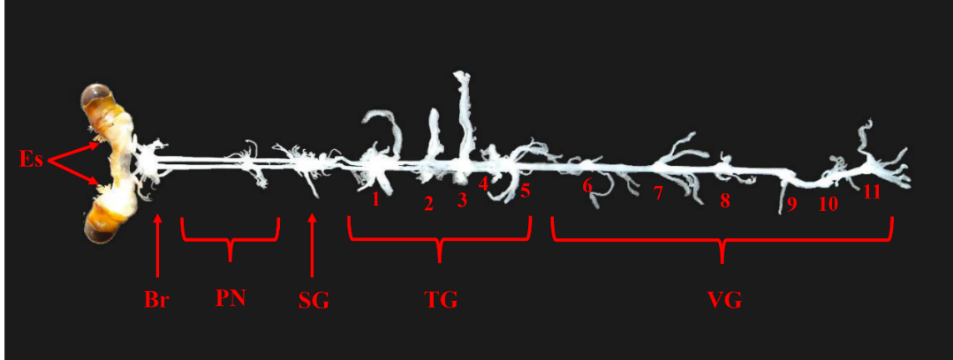
**

**Figure S1.** Anatomical map of the nervous system of *P. clarkii*. Es, eyestalk; Br, brain; PN, periosophageal nerve; SG, subesophageal ganglia; TG, thoracic ganglia; VG, ventral ganglia. There are 5 thoracic ganglia (1-5), which extends five pairs of walking leg nerves, there are 6 ventral ganglia (6-11), which extends five pairs of swimming foot and multiple pairs of tail nerves.
