# Supplementary material for: Molecular Characterization and Functional Study of Insulin-Like Androgenic Gland Hormone Gene in the Red Swamp Crayfish, Procambarus clarkii
Source: Genes (Basel). 2019 Aug 26;10(9):645. doi: 10.3390/genes10090645 (PMC6770367; doi:10.3390/genes10090645)
Supplement: Supplementary file 1 [file genes-10-00645-s001.zip › Supplementary Materials/Figure S2.docx]

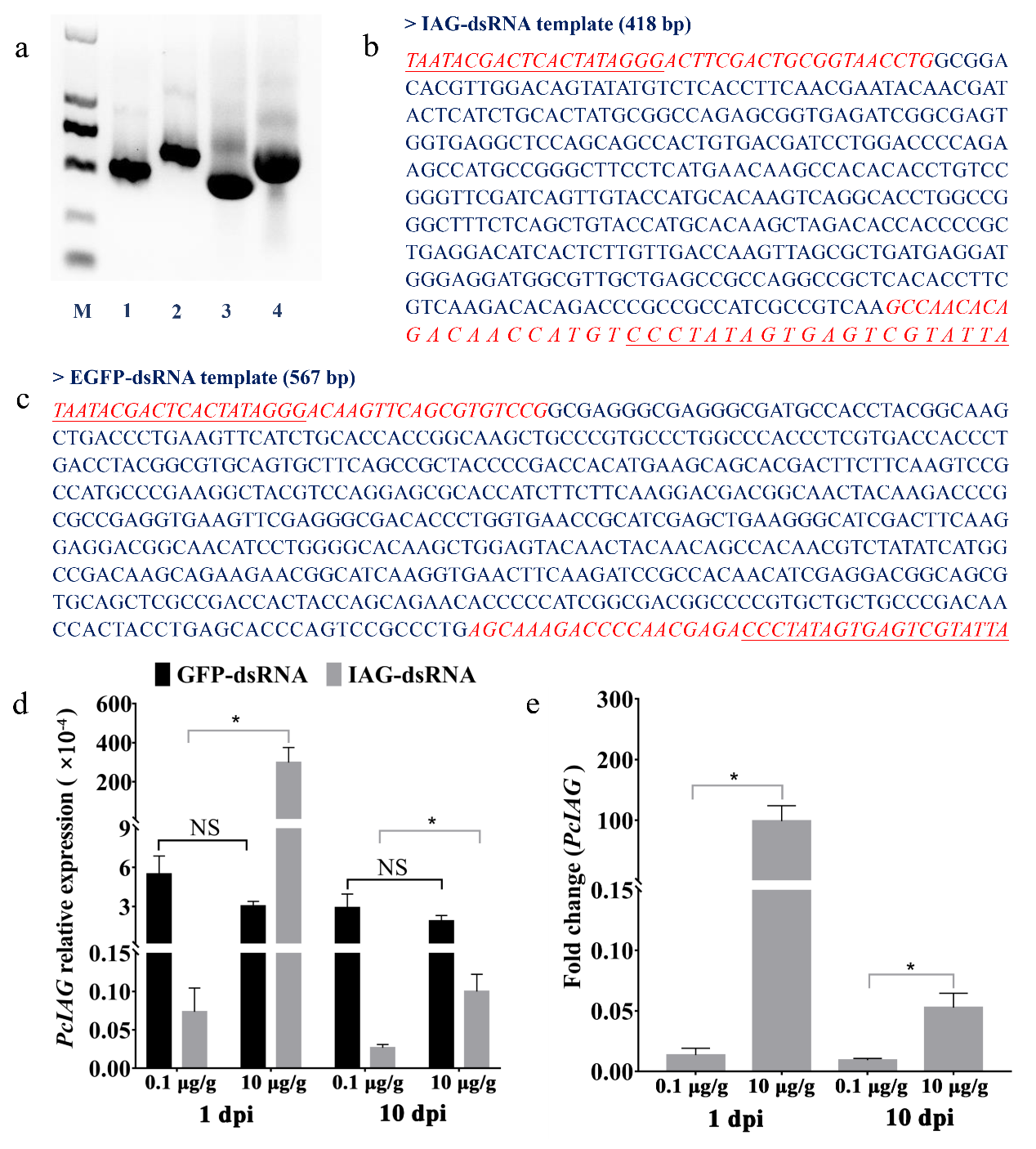


Figure S2. Detection of the dsRNA quality, DNA fragments and simplified analyses method explored for evaluating the effect of RNAi. a, Detection of the dsRNA quality with 1% agarose gel. M, DL2000 DNA Marker; 1, PcIAG-dsRNA template; 2, EGFP-dsRNA template; 3, PcIAG-dsRNA; 4, EGFP-dsRNA. b and c, DNA fragments sequence of template for synthesis into dsRNA. Specific primers were indicated by green italic font. The T7 promoter sequence was underlined. d and e, Simplified analyses method explored for evaluating the effect of RNAi based on the data randomly. d, The method evaluating the effect of RNAi by presenting *PcIAG* relative expression after injection of PcIAG-dsRNA and EGFP-dsRNA, respectively. *P* < 0.05 (*); NS, No significant. e, The method evaluating the effect of RNAi by calculating the trend of PcIAG expression fold change relationship between the same concentration of PcIAG-dsRNA group and the EGFP-dsRNA group at the same time point. *P* < 0.05 (*). d and e are obtained by processing the same raw data in two different data calculations, and the results showed that e was more simplified.
