# Supplementary material for: Molecular Characterization and Functional Study of Insulin-Like Androgenic Gland Hormone Gene in the Red Swamp Crayfish, Procambarus clarkii
Source: Genes (Basel). 2019 Aug 26;10(9):645. doi: 10.3390/genes10090645 (PMC6770367; doi:10.3390/genes10090645)
Supplement: Supplementary file 1 [file genes-10-00645-s001.zip › Supplementary Materials/Figure S3.docx]

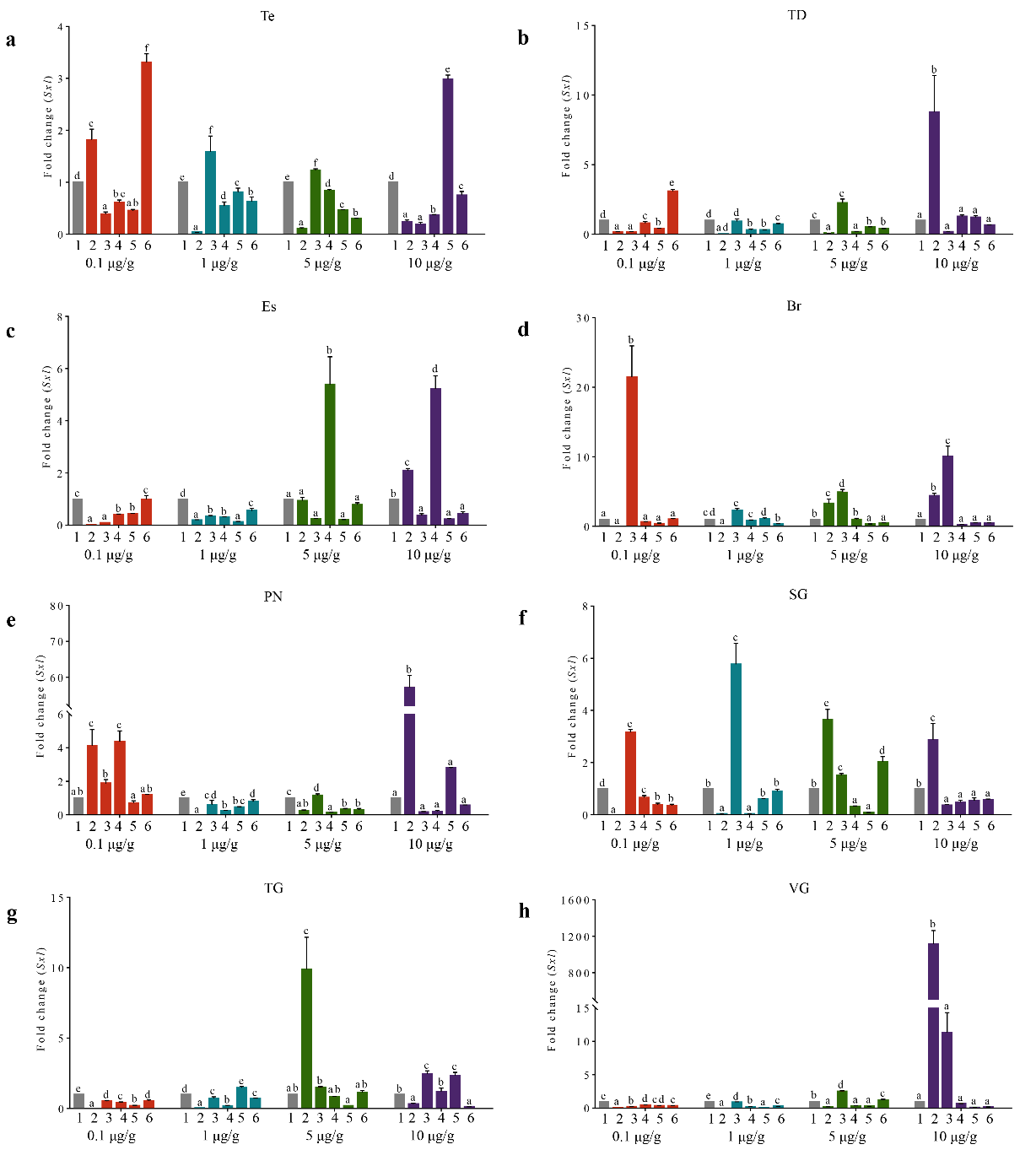


Figure S3. The change of *PcSxl* gene expression level in tissues at four doses of PcIAG-dsRNA. The column “1” in the figure represent the *PcSxl* expression after injection of EGFP-dsRNA. The columns “2”, “3”, “4”, “5” and “6” in the figure represent the *PcSxl* expression at different time points 1, 3, 7, 10 and 14 dpi after injection of PcIAG-dsRNA, respectively. a, Testis (Te); b, Testicular ducts (TD); c, Eyestalk (Es); d, Brain (Br), e, Periosophageal nerve (PN); f, Subesophageal ganglia (SG); g, Thoracic ganglia (TG); f, Ventral ganglia (VG). Different lower-case letters indicated significant differences among 1, 3, 7, 10 and 14 dpi for the same dsRNA concentration (*P* < 0.05).
