# Supplementary material for: Molecular Characterization and Functional Study of Insulin-Like Androgenic Gland Hormone Gene in the Red Swamp Crayfish, Procambarus clarkii
Source: Genes (Basel). 2019 Aug 26;10(9):645. doi: 10.3390/genes10090645 (PMC6770367; doi:10.3390/genes10090645)
Supplement: Supplementary file 1 [file genes-10-00645-s001.zip › Supplementary Materials/Table S1.docx]

Table S1. Primers used in this experiment

| Primer name | Use of primers | Sequence (5'-3') | Position | Expected size (bp) | Annealing temperature (ºC) |
| --- | --- | --- | --- | --- | --- |
| PcIAGF | Gene cloning | CTGCGGTAACCTGGCGGACACG | 191-212 | About 500 | 62 |
| PcIAGR |  | GAGCAAGGCGCCGTCCTCCGG | 707-687 |  |  |
| PcIAG5ʹR1 (Kit) |  | GATTACGCCAAGCTTGGTCCAGGATCGTCACAGTG |  | About 200 |  |
| PcIAG5ʹR2 (Kit) |  | GATTACGCCAAGCTTCTCACCGCTCTGGCCGCAT |  |  |  |
| PcIAG5ʹR1 |  | GTATCGTTGTATTCGTTGAAGGTGA |  | About 300 | 60 |
| PcIAG5ʹR2 |  | ACCGCAGTCGAAGTCCACCAGA |  |  |  |
| PcIAG5ʹR3 |  | CAGTACGAGGAGGATGGCAGC |  |  |  |
| PcIAG3ʹF1 |  | GCCGTCAAGCCAACACAGAC |  | About 200 |  |
| PcIAG3ʹF2 |  | CAGGACCTGCGTCCTGGAG |  |  |  |
| oligodT-anchor |  | GACCACGCGTATCGATGTCGACTTTTTTTTTTTTTTTTV |  |  |  |
| anchor |  | GACCACGCGTATCGATGTCGAC |  |  |  |
| PcIAG-dsF | RNAi | TAATACGACTCACTATAGGGACTTCGACTGCGGTAACCTG | 185-203 | 418 | 67 |
| PcIAG-dsR |  | TAATACGACTCACTATAGGGACATGGTTGTCTGTGTTGGC | 601-582 |  |  |
| EGFP-dsF |  | TAATACGACTCACTATAGGGACAAGTTCAGCGTGTCCG |  | 566 | 63 |
| EGFP-dsR |  | TAATACGACTCACTATAGGGTCTCGTTGGGGTCTTTGCT |  |  |  |
| PcIAG-qF | qPCR | CAGCAGCCACTGTGACGAT | 304-322 | 170 | 64 |
| PcIAG-qR |  | GATGTCCTCAGCGGGGTGG | 473-455 |  |  |
| PcSxl-qF |  | AGACTGGCTATTCGTTTGG |  | 112 | 59 |
| PcSxl-qR |  | TACTTGATGCGTTTATGCTG |  |  |  |
| 18S-RNA |  | TATACGCTAGTGGAGCTGGAA |  | 147 | 59 |
| 18S-RNA |  | GGGGAGGTAGTGACGAAAAAT |  |  |  |

Note: The T7 promoter sequence was underlined. The position information is based on the sequence of KT343750.1 in Genbank.
