# Supplementary material for: Molecular Characterization and Functional Study of Insulin-Like Androgenic Gland Hormone Gene in the Red Swamp Crayfish, Procambarus clarkii
Source: Genes (Basel). 2019 Aug 26;10(9):645. doi: 10.3390/genes10090645 (PMC6770367; doi:10.3390/genes10090645)
Supplement: Supplementary file 1 [file genes-10-00645-s001.zip › Supplementary Materials/Table S2.docx]

Table S2. Annotation of the *IAG* used in the present study

| Accession number | Organism | name | Preproprotein size (aa) |
| --- | --- | --- | --- |
| / | *Procambarus clarkii* | Insulin-like androgenic gland factor | 210 |
| AUL52689.1 | *Procambarus virginalis* | Insulin-like androgenic gland hormone | 210 |
| AUL52688.1 | *Procambarus fallax* | Insulin-like androgenic gland hormone | 210 |
| ADK46885.1 | *Portunus pelagicus* | Insulin-like androgenic gland factor | 176 |
| ART29878.1 | *Portunus trituberculatus* | Androgenic gland hormone | 146 |
| ASA45642.1 | *Chaceon quinquedens* | Insulin-like androgenic gland hormone | 151 |
| AOE46694.1 | *Eriocheir sinensis* | Insulin-like androgenic gland factor | 151 |
| AFY09905.1 | *Scylla paramamosain* | Insulin-like androgenic gland factor | 153 |
| AEI72263.1 | *Callinectes sapidus* | Insulin-like androgenic gland factor | 153 |
| BAC57013.1 | *Porcellio dilatatus* | Androgenic gland hormone precursor | 146 |
| BAC57012.1 | *Porcellio scaber* | Androgenic gland hormone precursor | 145 |
| BAA86893.1 | *Armadillidium vulgare* | Androgenic gland hormone | 144 |
| AHY99679.1 | *Sagmariasus verreauxi* | Insulin-like androgenic gland specific factor | 158 |
| AIM55892.1 | *Jasus edwardsii* | Insulin-like androgenic gland hormone | 154 |
| ACJ38227.1 | *Macrobrachium rosenbergii* | Insulin-like androgenic gland specific factor | 173 |
| AHZ34725.1 | *Macrobrachium vollenhovenii* | Insulin-like androgenic gland hormone | 176 |
| BAJ78349.1 | *Macrobrachium lar* | Insulin-like androgenic gland factor | 185 |
| AHA33389.1 | *Macrobrachium nipponense* | Insulin-like androgenic gland factor | 175 |
| ACD91988.1 | *Cherax destructor* | Insulin-like androgenic gland factor | 176 |
| ABH07705.1 | *Cherax quadricarinatus* | Insulin-like androgenic gland factor | 176 |
| ASM94212.1 | *Pandalus platyceros* | Insulin-like androgenic gland hormone | 167 |
| BAJ84109.1 | *Palaemon pacificus* | Insulin-like androgenic gland factor | 177 |
| BAJ84108.1 | *Palaemon paucidens* | Insulin-like androgenic gland factor | 173 |
| AFU60547.1 | *Penaeus chinensis* | Insulin-like androgenic gland hormone isoform 1 | 169 |
| ADA67878.1 | *Penaeus monodon* | Insulin-like androgenic gland hormone precursor | 176 |
| BAK20460.1 | *Penaeus japonicus* | Insulin-like androgenic gland factor | 170 |
